# Evaluation of Factors Predicting the Effectiveness of Regorafenib in the Treatment of Metastatic Colorectal Cancer

**DOI:** 10.5152/eurasianjmed.2022.21162

**Published:** 2022-10-01

**Authors:** Senar Ebinç, Zeynep Oruç, Zuhat Urakçi, Ziya Kalkan, Muhammet Ali Kaplan, Mehmet Küçüköner, Abdurrahman Işikdoğan

**Affiliations:** 1 Department of Medical Oncology, Dicle University Faculty of Medicine, Diyarbakır, Turkey

**Keywords:** Colon, cancer, regorafenib

## Abstract

**Objective:**

Regorafenib is a multikinase inhibitor, the effectiveness of which was demonstrated in metastatic colorectal cancer. This study aimed to investigate the factors that could predict the effectiveness of regorafenib.

**Materials and Methods:**

This study retrospectively reviewed the clinical characteristics, tumor characteristics, and previous therapies in 62 patients who presented to our center between 2016 and 2020 and used regorafenib for metastatic colorectal cancer. The effects of the investigated variables on the response obtained with regorafenib use were evaluated.

**Results:**

This study included a total of 62 patients diagnosed with metastatic colorectal cancer, of whom 30 (48.4%) were males and 32 (51.6%) were females. Patients' median age at diagnosis was 49 years (18-68). Regorafenib therapy yielded a disease control rate of 64% [complete response = 0, partial response= 14 (28%), and stable disease = 18 (36%)]. Objective response was obtained in 28% of patients [complete response = 0 and partial response = 14 (28%)]. Progression-free survival was 4 months. The evaluation of the effects of patients' age, sex, performance status, previous treatments, metastatic sites, and RAS mutation status on the disease control rate and progression-free survival did not determine any positive or negative effects on progression-free survival. However, left-sided tumors had a positive effect on disease control rate (69.8% vs. 28.6%, *P* = .029). and previous use of cetuximab had a negative effect on disease control rate [76.5% vs. 37.5% (*P* = .007)].

**Conclusion:**

In our study, tumor localization and previous cetuximab use were found to be correlated with the disease control rate in patients on regorafenib. However, the need for novel biomarkers that will predict the effectiveness of regorafenib in metastatic colorectal cancer treatment persists.

**Main Points**

- Eastern Cooperative Oncology Group performance status, metastatic site, rechallenge therapy, RAS status, tumor localization, and type of targeted agent previously used (bevacizumab/cetuximab/panitumumab) had no effect on progression-free survival achieved with regorafenib in the treatment of metastatic colorectal cancer.
- Disease control rate (DCR) achieved with regorafenib was lower in patients who had received cetuximab before compared with those who had not.
- Left-sided tumors had better 3-month DCR with the use of regorafenib than right-sided tumors in multivariate analysis.

## Introduction

Colon cancer is the third most prevalent type of cancer worldwide. However, it constitutes the second most common cause of cancer-related mortality.^1^ Metastatic colorectal cancer (mCRC) treatment generally involves non-curative, palliative chemotherapy for most patients, although different scenarios exist.^2^ For the treatment of mCRC, 3 active conventional chemotherapy agents, namely, fluoropyrimidines, irinotecan, and oxaliplatin are used in various combinations. However, longer survival times can be achieved by the consecutive use of all active agents, rather than a single chemotherapy protocol.^3^ In mCRC, a better response can be obtained by the addition of bevacizumab, which is an anti-vascular endothelial growth factor (VEGF), to the treatment, regardless of the RAS mutation status.^4^ In the treatment of RAS wild-type mCRC, the addition of the anti-epidermal growth factor receptor (EGFR) agents cetuximab and panitumumab to chemotherapy improved survival.^5,6^ Regorafenib appears as an option in the case of progression in patients who previously used fluoropyrimidine, irinotecan, oxaliplatin-based therapies, anti-VEGF, and in RAS wild-type patients, anti-EGFR. The effectiveness of regorafenib, which is a multikinase inhibitor, was demonstrated in mCRC patients who received multiple therapies.^7^ In the literature, various predictors that might indicate the effectiveness of regorafenib have been investigated.^8^ However, no predictive factor has been identified to date. In this study, we aimed to investigate the factors predicting the effectiveness and tolerability of and the response to regorafenib in mCRC treatment.

## Materials and Methods

In this study, patients who presented to the Medical Oncology Clinic of Dicle University, Faculty of Medicine between 2016 and 2020 and who used regorafenib for a diagnosis of metastatic colorectal cancer were retrospectively screened. Data from a total of 62 patients could be acquired. Patients' data including age, sex, Eastern Cooperative Oncology Group performance status (ECOG PS), comorbidity, tumor histology, tumor localization (cecum, ascending colon, hepatic flexure, and transverse colon tumors were analyzed as right-sided and splenic flexure, descending colon, sigmoid colon, and rectum tumors were analyzed as left-sided), metastatic sites, RAS mutation status, treatments received before and after regorafenib, biological agents received before regorafenib (bevacizumab/panitumumab–cetuximab), starting dose of regorafenib, patient tolerability, and side effects associated with regorafenib were obtained from patient files. Whether or not tumor characteristics and clinical parameters predicted the response to regorafenib was analyzed. Treatment response was evaluated every 3 months based on clinical and radiological results. Radiological response was assessed according to the Response Evaluation Criteria in Solid Tumors.

## Statistics

Predictive Analytics Software (PASW) Statistics for Windows, Statistical Package for the Social Sciences v.18 (IBM Corp., Armonk, NY, USA) was used in the statistical analysis of the data. Descriptive statistics were used to evaluate patient characteristics and the frequencies of the parameters, Student's *t*-test was used for normally distributed numeric variables, and the Mann–Whitney *U* test was used for the analyses of non-normally distributed or non-parametric variables. In univariate analyses, the *t*-test, chi-square test, Fisher's exact, and Mann–Whitney *U* tests were used. A 95% CI and a *P*-value significance level of <.05 were adopted.

Thirteen clinical variables were identified based on previous studies. These were given as follows: ECOG PS (0-1/≥2), presence of comorbidity, tumor localization (right/left), RAS status (wild/mutant), metastatic localization (liver, lung, peritoneum, and bone), prior anti-EGFR (cetuximab–panitumumab), and anti-VEGF treatments (bevacizumab), line in which regorafenib was used (1-2/>2), and the state of having received rechallenge therapy prior to regorafenib. The Kaplan–Meier method (log-rank, Breslow, Tarone–Ware tests) and cox regression analysis were used for survival analysis. Overall survival (OS) was calculated as the duration of time from the diagnosis to mortality, metastatic OS as the duration of time from metastatic progression to mortality, and progression-free survival (PFS) for regorafenib as the duration of time from regorafenib initiation to progression while on regorafenib. Disease control status was defined as the sum of complete response (CR), partial response (PR), and stable disease (SD) at the third month of regorafenib therapy. The objective response was accepted as the sum of CR and PR in the third month of regorafenib therapy.

## Ethics consent

The study was approved by the research ethics committee of Dicle University, Faculty of Medicine (date/reference number: February 25, 2020/125). All analyses were performed in accordance with the principles of the Declaration of Helsinki.

## Results

Our study included a total of 62 patients diagnosed with metastatic colorectal carcinoma, of whom 30 (48.4%) were males and 32 (51.6%) were females. Patients’ median age at diagnosis was 49 years (18-68). At regorafenib initiation, 45 (72.6%) of our patients had an ECOG PS of 0-1, while 17 (27.4%) had an ECOG PS of 2-3. In total, 9 (14.5%) patients had at least 1 comorbidity; 50 (80.6%) patients had adenocarcinoma, 10 (16.1%) patients had mucinous adenocarcinoma, and 2 (3.2%) patients had signet ring-cell carcinoma histology. The tumor was localized on the right side in 9 (14.5%) patients and on the left side in 53 (85.5%) patients. Tumor localization in the colon were as follows: ascending colon in 5 (8.1%) patients, transverse colon in 4 (6.5%) patients, descending colon in 5 (8.1%) patients, sigmoid colon 27 (43.5%) patients, and rectum in 21 (33.9%) patients. Thirty-one (50%) patients had a wild type and 31 (50%) patients had mutant K/N-RAS. RAS status in right-sided tumors was wild in 3 (33.3%) patients and mutant in 6 (66.6%) patients. However, in left-sided tumors, 28 (52.8%) patients were RAS wild and 25 (47.2%) patients were RAS mutant. Of the patients included in the study, 36 (58.1%) were metastatic at diagnosis, while recurrent-metastatic disease developed after curative or palliative treatment in 26 (41.9%) patients. There were 41 (66.1%) patients who underwent curative or palliative surgery for the primary tumor and 18 (29%) patients who underwent radiotherapy for rectal cancer. Thirty (48.4%) patients who initially underwent complete resection received adjuvant chemotherapy. At the metastatic stage, 40 patients (64.5%) had liver metastasis, 19 (30%) had lung metastasis, 13 (21%) had bone metastasis, 3 (4.8%) had brain metastasis, and 16 (25.8%) had peritoneal metastasis. All patients had received prior fluoropyrimidine, oxaliplatin, and irinotecan as 3 active conventional chemotherapy agents for metastatic disease. As treatments prior to regorafenib, 54 (87.1%) patients had received bevacizumab, 19 (30.6%) had received cetuximab, and 10 (16.1%) had received panitumumab. Eighteen (29%) patients had received rechallenge therapy with one of the treatment regimens used in adjuvant therapy or at the metastatic stage before regorafenib. After regorafenib therapy, 15 (24.2%) patients received 1 line, 9 (14.5%) patients received 2 lines, 2 (3.2%) patients received 3 lines, and 1 (1.6%) patient received 4 lines of therapy. However, 35 (56.5%) patients could not receive any other treatment after regorafenib therapy.

Regorafenib was used as the second-line treatment in 2 (3.2%) patients, third-line treatment in 41 (66.1%) patients, and fourth-line treatment (including adjuvant treatment) in 19 (30.6%) patients. The starting dose of regorafenib was 80 mg/day in 9 (14.5%) patients, 120 mg/day in 7 (11.3%) patients, and 160 mg/day in 46 (74.2%) patients. The treatment was discontinued due to regorafenib intolerance in 13 (20.9%) patients. The drug dose was reduced in 19 (30.6%) patients and the drug was interrupted in 1 (1.6%) patient due to side effects. The comparison of the starting doses of regorafenib [standard dose (160 mg/day) or low dose (80-120 mg/day)] did not determine a statistically significant difference between the 2 groups in terms of drug tolerance or the development of side effects (*P* = .088).

A response could not be evaluated in 12 (19.4%) patients initiated on regorafenib due to the discontinuation of the drug and the short duration of treatment. The effectiveness of regorafenib was evaluated in 50 patients who were able to use the drug regularly and in whom responses could be evaluated. Of these 50 patients, 14 (28%) showed PR, 18 (36%) showed SD, and 18 (36%) showed progression. None of the patients showed CR. In patients who used regorafenib regularly, the DCR was 64% (n = 32) [CR = 0, PR = 14 (28%), SD = 18 (36%)]. The objective response rate (ORR) was 28% (n = 14) [CR = 0, PR = 14 (28%)]. The group in which disease control was achieved with regorafenib and that in which disease control was not achieved with regorafenib were compared in terms of age, sex, ECOG PS, comorbidity, tumor localization, RAS mutation status, metastatic site, previously received biological agents, line in which regorafenib was used and rechallenge therapy (Table 1). Of these parameters, tumor localization and previous use of cetuximab were determined to be correlated with the DCR. While the DCR was 28.6% for tumors localized on the right side, it was 69.8% for tumors localized on the left side. This difference had borderline statistical significance (*P* = .05) in univariate analysis, but there was a significant difference in multivariate analysis (*P = *.029). Previous use of cetuximab was determined to have an unfavorable prognostic effect on the DCR. The DCR was lower at 37.5% in patients who had received cetuximab, as opposed to 76.5% in patients who had not received cetuximab (*P* = .007).

The median overall survival time from the onset of metastatic disease was 27 months (95% CI: 22.9-31), and the median OS from regorafenib initiation was 6 months (95% CI: 4.4-7.5). The median PFS time obtained with regorafenib was 4 months (95% CI: 2.9-5.0). The median PFS was 3 months (95% CI: 2.3-3.6) for the arm that had received cetuximab and 5 months (95% CI: 2.7-7.2) for the arm that had not. There was no statistically significant difference between the 2 groups in terms of PFS [hazard ratio (HR): 1.60 (95% CI 0.80-3.21), *P* = .17]. When evaluated in terms of OS, the arm that had received cetuximab had a median OS of 10 months (95% CI: 2.1-17.8), while the arm that had not received cetuximab had a median OS of 5 months (95% CI: 3.8-6.1) (log rank, *P* = .63), [HR: 1.86 (95% CI 0.99-3.51), *P* = .053]. When compared with regard to PFS, median PFS with regorafenib was 3 months in those who had received rechallenge therapy, while it was 5 months in those who had not. There was no statistically significant difference between the 2 groups (log rank, *P* = .18). In the evaluation of the prognostic factors that could predict PFS, none of the 13 variables showed a statistically significant effect on the PFS achieved with regorafenib (Table 2).

Regorafenib was associated mostly with grade 1-2 side effects. As grades 3-4 side effects, fatigue was encountered in 11 (17.7%) patients, hypertension in 2 (3.2%) patients, and hand-foot syndrome in 5 (8.1%) patients. The observed side effects are specified in detail in Table 3.

In this study, we investigated the factors that influence treatment effectiveness, tolerability, and treatment response in patients who received regorafenib in the treatment of mCRC. In our study, an OS of 6 months, PFS of 4 months, and DCR of 64% were achieved with regorafenib use. Many studies have previously investigated the effectiveness of regorafenib in the treatment of mCRC. In the CONCUR study, regorafenib was compared with placebo in mCRC patients who received at least 2 treatment lines and regorafenib was found to offer OS and PFS advantage in comparison with placebo. In the CONCUR study, an OS of 8.8 months and a PFS of 3.2 months were obtained with regorafenib use.^9^ In our study, the median OS and PFS obtained with regorafenib were 6 and 4 months, respectively. Overall survival was shorter in our study. In evaluating this result, it must be noted that prospective clinical studies include selected patient groups with favorable ECOG PS and clinical characteristics and a low tumor burden. The CONCUR study included patients with an ECOG PS of 0-1.^9^ In our study, 27% of the patients had an ECOG PS > 1. However, the PFS obtained in our study was consistent with the CONCUR study (4 months vs. 3.2 months). In the placebo-controlled, phase-3 CORRECT study, the disease control rate associated with regorafenib was found as 41%.^7^ In our study, the DCR was 64% (n = 32). The DCR achieved in our study was better when compared with the CORRECT study.

The localization and molecular characteristics of the tumor play a guiding role in the treatment decision regarding the first-line mCRC treatment. RAS status is a predictive factor in the selection of a biological agent (anti-VEGF/anti-EGFR).^4,6,10^ Meanwhile, a factor that can predict effectiveness in regorafenib therapy has not yet been defined. However, various biomarkers have been examined in the literature. In the retrospective analysis of the CORRECT study, markers, such as circulating DNA levels, RAS status were examined. However, the association of these markers with regorafenib could not be clearly shown.^8^ Various studies have been conducted on the treatment line in which regorafenib would be used and the parameters indicating its effectiveness. In the PREVIUM study, the use of second-line, single-agent regorafenib after FOLFOXIRI–bevacizumab therapy in the treatment of RAS mutant mCRC was tested. Unfortunately, this study did not obtain favorable results regarding the selection of regorafenib in earlier lines of therapy.^11^ In our study, 2 patients used regorafenib as the second-line treatment, while other patients used it as the third- or later-line treatment. When patients who had received rechallenge therapy with treatments given before regorafenib as adjuvant therapy or after regorafenib were compared with patients who had not, PFS achieved with regorafenib were 3 and 5 months, respectively. However, there was no statistically significant difference between the 2 groups (log rank, *P* = .18). In the CORRECT study; ECOG PS, time from diagnosis, number of metastases, and presence of liver metastasis were evaluated as prognostic factors, while in the REBECCA study, a prognostic scoring was applied by adding the starting dose of regorafenib and K-RAS mutation status to these factors.^8,12^ With the prognostic model devised in the REBECCA study, OS was determined as 9.2 months in the low-risk group and 2.5 months in the high-risk group.^12^ In our study, as potential predictors of PFS; age, sex, ECOG PS, presence of comorbidity, metastatic localization, RAS status, anti-EGFR agents used before regorafenib, anti-VEGF use, treatment line in which regorafenib was used (1,2/>2), and the state of having received rechallenge therapy were evaluated. None of these variables included in our study predicted the PFS obtained with regorafenib. With regard to the disease control rate, the 3-month disease control rate obtained with regorafenib was lower in patients who had received cetuximab before regorafenib than in those who had not (37.5% vs. 76.5%). This difference was statistically significant (*P* = .007). In a study conducted by Shitara K. and colleagues, cetuximab use following regorafenib and regorafenib use following cetuximab were compared in patients who progressed after fluoropyrimidine/oxaliplatin/irinotecan-based chemotherapies in the treatment of mCRC. This study obtained an OS of 17.4 months with cetuximab use following regorafenib, whereas an OS of 11.6 months was obtained with regorafenib use following cetuximab.^13^ In our study, patients who had received cetuximab before regorafenib were not significantly different than those who had not in terms of PFS [HR: 1.60 (95% CI: 0.80-3.21), *P* = .17] and OS [HR: 1.86 (95% CI: 0.99-3.51), *P* = .053]. However, the 3-month DCR was better in the arm that had not received cetuximab (37.5% vs. 76.5%). Previous in vitro studies proposed that regorafenib and cetuximab take effect through similar common mechanisms.^14^ We reason that the lower regorafenib effectiveness determined in the patients in our study who had received cetuximab can be associated with the resistance that may have developed in these common mechanisms. Studies are needed to confirm this hypothesis in larger populations. In the study of Sung et al.^15^ it was observed that the efficacy of regorafenib was better in tumors located in the left colon. In our study, there was no difference in PFS in the use of regorafenib according to tumor localization. However, in multivariate analysis, left-sided tumors had a better 3-month DCR than right-sided tumors (69.8% vs. 28.6%, *P* = .029).

The side effects associated with regorafenib in the treatment of mCRC were evaluated in 2 large studies, namely the CONSIGN and CORRELATE studies. The most common side effects included fatigue, hand-foot syndrome, and diarrhea.^16,17^ Accordingly, the most common side effect encountered in our study was fatigue, followed by hand–foot syndrome. Other side effects were consistent with the literature^16,17^ (Table 3). In the CONSIGN study, regorafenib use was interrupted in 60% of the patients, while the dose was reduced in 49%.^15^ In the CORRELATE study, drug use was interrupted in 50% of the patients and the dose was reduced in 47% of the patients.^17^ In our study, the treatment was continued with dose reduction in 19 (30.6%) patients. Meanwhile, in 10 (16.1%) patients, the dose was reduced first and the drug was discontinued when the patients failed to tolerate the treatment.

The ReDOS study emphasized that initiating regorafenib therapy by dose escalation in the first cycle would be beneficial in terms of drug tolerance.^18^ In our study, with regard to intolerance, there was a difference approaching statistical significance between patients who started the treatment with standard-dose regorafenib (160 mg/day) and patients who started the treatment with a lower dose (80-120 mg/day), favoring those who started the treatment with a lower dose (*P* = .088).

The limitations of our study include conditions such as its retrospective design, the low number of patients, the heterogeneity of the patient population, and the heterogeneity of treatment lines received before and after regorafenib. In conclusion, knowledge of the factors that can predict the effectiveness of regorafenib, the contribution of which was shown in third-line therapy in the treatment of mCRC, would facilitate the approach to the patient. In this study, we determined that the effectiveness of regorafenib was independent of factors such as ECOG PS, metastatic site, rechallenge therapy, RAS status and that the disease control rate achieved with regorafenib was lower in patients who had received cetuximab before compared with those who had not. Left-sided tumors had better 3-month DCR than right-sided tumors in multivariate analysis. Novel biomarkers that will indicate the effectiveness and side effects of regorafenib are needed.

## Figures and Tables

**Table 1.** Clinical Features of All Patients and Variables Investigated for Disease Control Rate in Patients Who Have Used Regorafenib

		**No, n (%)**	**Yes, n (%)**	***P***
		**All Patients (n = 62, %)**	**Disease Control Rate (n = 50)**		
**Age**						.33
(median, range)		49 (18-68)	46.5 (23-63)	48.5 (18-68)	
**Gender**					.86
	Male	30 (48.4)	8 (34.8)	15 (65.2)	
	Female	32 (51.6)	10 (37)	17 (63)	
**ECOG PS**					.64
	0-1	45 (72.6)	14 (37.8)	23 (62.2)	
	>1	17 (27.4)	4 (30.8)	9 (69.2)	
**Co-morbidities**					.20
	Yes	9 (14.5)	4 (57.1)	3 (42.6)	
	No	53 (85.5)	14 (32.6)	29 (67.4)	
**Tumor site**					**.035**
	Right	9 (14.5)	5 (71.4)	2 (28.6)	
	Left	53 (85.5)	13 (30.2)	30 (69.8)	
**RAS status**					.077
	Wild	31 (50)	12 (48)	13 (52)	
	Mutant	31 (50)	6 (24)	19 (76)	
**Liver metastasis**					.76
	Yes	40 (64.5)	12 (37.5)	20 (65.2)	
	No	22 (35.5)	6 (33.3)	12 (66.7)	
**Lung metastasis**					.36
	Yes	19 (30.6)	5 (27.8)	13 (72.2)	
	No	43 (69.4)	13 (40.6)	19 (59.4)	
**Bone metastasis**					.36
	Yes	13 (21)	4 (50)	4 (50)	
	No	49 (79)	14 (33.3)	28 (66.7)	
**Peritoneal metastasis**					.064
	Yes	16 (25.8)	7 (58.3)	5 (41.7)	
	No	46 (74.2)	11 (28.9)	27 (71.1)	
**Bevacizumab treatment**					.20
	Yes	54 (87.1)	14 (32.6)	29 (67.4)	
	No	8 (12.9)	4 (57.1)	3 (42.9)	
**Cetuximab treatment**					**.007**
	Yes	19 (30.6)	10 (62.5)	6 (37.5)	
	No	43 (69.4)	8 (23.5)	26 (76.5)	
**Panitumumab treatment**					.39
	Yes	10 (16.1)	1 (16.1)	5 (83.3)	
	No	52 (83.9)	17 (38.6)	27 (61.4)	
**Regorafenib treatment line**					.79
	2-3rd	43 (69.4)	13 (37.1)	22 (62.9)	
	>3rd	19 (30.6)	5 (33.3)	10 (66.7)	
**Rechallenge treatment**					
	Yes	18 (29)			
	No	44 (71)			
**Total**		62 (100)	18 (36)	32 (64)	

ECOG PS, Eastern Cooperative Oncology Group performance status.

**Table 2.** Univariate and Multivariate Analysis Results for Progression-Free Survival and Disease Control

**Univariate Analysis**			**Univariate Analysis**			**Multivariate Analysis**		
**HR**	**95% CI**	***P***	**HR**	**95% CI**	***P***	**HR**	**95% CI**	***P***
	**Progression-Free Survival**				**Disease Control**				
	**ECOG PS (0-1, >1)**	1.48	6.86-3.21	.31	1.37	0.35-5.29	.64			
	**Co-morbidities (no/yes)**	0.73	0.25-2.09	.55	0.36	0.71-1.84	.22			
**Tumor site (right, left)**	0.94	0.36-2.48	.91	5.76	0.98-33.67	**.05**	8.52	1.24-58.22	**.029**
**RAS status (wild/mutant)**	0.71	0.35-1.45	.35	2.92	0.87-9.77	.82			
**Liver metastasis**	1.36	0.65-2.83	.40	0.83	0.24-2.80	.76			
**Lung metastasis**	1.10	0.53-2.29	.79	1.77	0.51-6.20	.36			
**Bone metastasis**	1.36	0.59-3.16	.46	0.50	0.10-2.30	.37			
**Peritoneal metastasis**	1.25	0.58-2.70	.56	0.29	0.76-1.11	.07			
**Bevacizumab treatment**	0.99	0.38-2.60	.99	2.76	0.54-14.05	.22			
**Cetuximab treatment**	1.6	0.80-3.21	.17	0.18	0.05-0.66	**.01**	0.14	0.036-0.58	**.006**
**Panitumumab treatment**	0.85	0.29-2.50	.77	3.14	0.33-29.31	.31			
**Regorafenib treatment line (2-3/>3)**	1.4	0.65-3.08	.38	1.18	0.33-4.22	.79			
**Rechallenge treatment**	1.6	0.71-3.68	.24	1.01	0.28-3.68	.97			

ECOG PS, Eastern Cooperative Oncology Group performance status; HR, hazards ratio.

**Table 3.** Adverse Events Due to Regorafenib

	**Any Grade (n, %)**	**Grade 1/2**	**Grade ≥ 3**
**Hypertension**	5 (8.1)	3 (4.8)	2 (3.2)
**Palmar-plantar erythrodysesthesia**	25 (40.1)	20 (32.2)	5 (8.1)
**Dermatologic**	8 (12.9)	7 (11.3)	1 (1.6)
**Fatique**	39 (62.7)	28 (45)	11 (17.7)
**Hypothyroidism**	2 (3.2)	2 (3.2)	0
**Nausea/vomiting**	16 (25.7)	15 (24.1)	1 (1.6)
**Diarrhea**	10 (16.1)	7 (11.3)	3 (4.8)
**Anemia**	12 (19.4)	12 (19.4)	0
**Lymphocytopenia**	3 (4.8)	3 (4.8)	0
**Thrombocytopenia**	13 (21)	12 (19.4)	1 (1.6)
**Hepatic**	12 (11.4)	11 (17.7)	1 (1.6)
